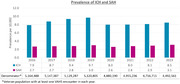# Validation of ICD‐10 diagnosis codes for identification of veterans with intracerebral hemorrhage and subarachnoid hemorrhage using clinical notes in the United States Veterans Affairs Healthcare System

**DOI:** 10.1002/alz.086944

**Published:** 2025-01-09

**Authors:** Byron J. Aguilar, Vanesa Carlota Andreu Arasa, Peter J Morin, Ying Wang, Brant Mittler, Dan Berlowitz, Myriam Abdennadher, Joel Reisman, Henry W Querfurth, Raymond Zhang, Amir Abbas Tahami Monfared, Quanwu Zhang, Weiming Xia

**Affiliations:** ^1^ The Bedford VA Research Corporation, Inc., Bedford, MA USA; ^2^ Geriatric Research Education & Clinical Center, VA Bedford Healthcare System, Bedford, MA USA; ^3^ Boston University Chobanian & Avedisian School of Medicine, Boston, MA USA; ^4^ Neuroradiology Service, VA Boston Healthcare System, Boston, MA USA; ^5^ Wentworth Institute of Technology, Boston, MA USA; ^6^ Geriatric Research Education & Clinical Center, VA South Texas Healthcare System, San Antonio, TX USA; ^7^ University of Massachusetts Lowell, Lowell, MA USA; ^8^ Center for Healthcare Organization & Implementation, VA Bedford Healthcare System, Bedford, MA USA; ^9^ Tufts Medical Center, Tufts University, Boton, MA USA; ^10^ Alzheimer’s Disease & Brain Health, Eisai Inc., Nutley, NJ USA; ^11^ McGill University, Montreal, QC Canada

## Abstract

**Background:**

Cerebral amyloid angiopathy (CAA) is a significant contributor to hemorrhagic stroke, notably lobar intracerebral hemorrhage (ICH) and convexity subarachnoid hemorrhage (SAH). This study describes the natural occurrence of ICH and SAH events among veterans, including those with AD, within the United States Veterans Affairs Healthcare System (VAHS).

**Method:**

The VAHS database was evaluated to identify ICD‐10 codes for ICH (I61.x) and SAH (I60.x) from 2015‐2023. A subsample of veterans with AD was identified based on 1 qualifier (AD diagnostic code or clinical note); a sensitivity analysis included veterans with ≥2 AD qualifiers, ≥30 days apart. Two‐thousand veterans were randomly selected from the ICH/SAH sample for validation of diagnostic coding using clinical notes. The positive predictive value (PPV) of ICH/SAH diagnostic codes was determined using notes from 100 randomly selected cases.

**Result:**

A total of 23,539 and 7,822 veterans were identified using ICD‐10 codes for ICH and SAH, respectively, out of 4‐5 million veterans receiving care annually. The ICH/SAH sample was 93/95% male, 65/69% white, with a mean age of 70 years. Approximately 14% and 4/5% of veterans with ICH/SAH had AD based on 1 and 2 AD qualifiers, respectively. From 2016‐2023, the yearly prevalence of ICH and SAH was approximately 8‐10 and 3/10,000 patients, respectively (**Figure**). Approximately 61/68% of veterans in the ICH/SAH sample were identified from outpatient visits only and 39/32% were identified from VAHS inpatient stays with or without outpatient medical records. Among 2,000 randomly selected cases for coding validation, 98% had notes available within ±14 days of the ICD coding; among these, 55/60% carried an ICH/SAH keyword. Brain MRI records were found in one‐third of ICH/SAH cases. Review of 2,000 clinical notes corresponding to 100 randomly selected cases found documentation of ICH in 80% (PPV) of veterans with an ICH diagnostic code and 100% with an SAH diagnostic code.

**Conclusion:**

Yearly prevalence of ICH/SAH (2016‐2023) was 0.08‐0.1%/0.03% in US veterans with 14% of the total cases carrying ≥1 AD identifier. Among cases with clinical notes for ICH and SAH, PPVs were 80% and 100%, respectively.